# Pathogenesis of renal involvement in primary Sjögren’s disease: convergence of multifactorial mechanisms on immune dysregulation

**DOI:** 10.3389/fimmu.2026.1790828

**Published:** 2026-05-08

**Authors:** Jingya Yang, Yingchun Cui, Xiaoying Bai, Qiaoyan Guo, Wenpeng Cui, Feng Xu

**Affiliations:** Department of Nephrology and Rheumatology Immunology, The Second Hospital of Jilin University, Changchun, Jilin, China

**Keywords:** B cells, CD4+ T cells, ectopic germinal center, genetic susceptibility, IFN-I, primary Sjögren’s disease, renal involvement

## Abstract

Primary Sjögren’s disease (pSjD) is a chronic systemic autoimmune disease in which immune-mediated pathology is not confined to exocrine glands but also affects multiple organ systems. Renal involvement constitutes a clinically meaningful manifestation of pSjD and may exert a considerable impact on disease progression, prognosis, and treatment selection. However, the pathogenic basis of renal injury in pSjD is still incompletely understood, and existing therapeutic approaches remain largely empirical. Available studies suggest that renal involvement in pSjD arises from the interaction of multiple determinants, including inherited susceptibility, environmental factors, and endocrine dysregulation. At the core of these pathogenic mechanisms lies persistent activation of the type I interferon (IFN-I) system and immune dysregulation driven by excessive responses of T and B lymphocytes. Such immune abnormalities favor sustained autoantibody production and ectopic germinal center (EGC) formation, thereby amplifying autoimmune inflammation and promoting renal structural and functional injury. Here, this review integrates relevant literature to examine the multifactorial pathogenic mechanisms described above and to systematically elucidate how these mechanisms drive autoimmune responses through diverse immune cell populations. In addition, the potential application prospects of relevant novel targeted therapeutic strategies in pSjD-related renal damage are discussed.

## 1 Introduction

Sjögren’s disease (SjD) is a systemic autoimmune disorder mainly characterized by infiltration of lymphocytes and plasma cells into exocrine glands. The disease primarily affects the salivary and lacrimal glands, leading to the characteristic clinical features of dryness in the eyes and mouth, accompanied by multiple autoantibodies in patient serum (1). When SjD occurs alongside other systemic autoimmune diseases meeting classification criteria, such as lupus or rheumatoid arthritis, it is termed associated Sjögren’s disease (aSjD). By contrast, SjD occurring without other systemic autoimmune diseases is referred to as primary Sjögren’s disease (pSjD) (2). pSjD is a systemic condition with an estimated prevalence of 0.01% to 0.1% in the general population. The disorder most commonly affects adults aged 40 to 50, with women being disproportionately affected.

Beyond the classic impairment of the salivary and lacrimal glands, pSjD can involve multiple organ systems. It may affect the hematologic, musculoskeletal, respiratory, and renal systems (1). Renal involvement is a crucial factor influencing prognosis in pSjD, affecting roughly 4% to 30% of patients during the disease course (3). Tubulointerstitial nephritis (TIN) is the most common renal manifestation in pSjD, primarily resulting from extensive infiltration of lymphocytes and plasma cells into the renal tubules and interstitium. These alterations resemble histopathological features observed in affected exocrine tissues, such as the salivary glands, ultimately leading to tubular atrophy and interstitial fibrosis. Polyclonal B-cell activation, a hallmark of pSjD, can trigger cryoglobulinemia, which may give rise to membranoproliferative glomerulonephritis (MPGN), the second most common renal pathology in this condition (4). In addition, extensive infiltration of lymphocytes and plasma cells may result in nodular lymphoid aggregates containing germinal centers (GCs), further aggravating renal injury (5). Studies indicate that patients with renal involvement exhibit a 15-year mortality of about 25% and a 28-year mortality approaching 80%. Within this group, individuals with glomerulonephritis (GN) face a higher risk of death than those with TIN, with a 15-year mortality of roughly 60% (6). The relatively high prevalence of renal complications in pSjD, along with their strong link to adverse outcomes, highlights the need for a systematic understanding of the underlying pathogenesis.

The mechanisms underlying renal involvement in pSjD have not yet been fully elucidated. Current evidence suggests that renal manifestations in pSjD arise from the interaction of genetic predisposition, immune dysregulation, environmental influences, and endocrine factors (**Table 1** summarizes the core pathogenic mechanisms driving these processes). In this review, we synthesize research advances in the classification and pathogenesis of renal involvement in pSjD, with reference to the updated International Classification of Diseases. We further provide a systematic overview of the proposed pathogenic mechanisms to inform future research and advance the development of targeted therapeutic strategies.

**Table 1:** Role of different categories in the pathogenesis of renal involvement in pSjD.

Category	Factor/Molecule	Effect	Refs.
Genes	HLA-DRB1*03:01	Core 8.1 AH risk allele. Encodes an HLA-II molecule with high affinity for SSA/SSB peptides, driving loss of tolerance and providing the genetic foundation for humoral and cellular renal injury.	(7–10)
	HLA-DRB1*15:01	A potential risk allele for pSjD-MN, linked via PLA2R positivity.	(11–13)
	TNF2	Pro-inflammatory variant that increases TNF-α production, conferring risk for proteinuria and dRTA in pSjD.	(14)
	IRF5/STAT4	Correlates with anti-SSA/SSB positivity; synergistically activates IFN-I, driving the systemic interferon signature in pSjD.	(15, 16)
	BLK	Regulates BCR signaling and NF-κB activation, enabling autoreactive B-cell escape and promoting autoantibody production.	(17, 18)
	CXCR5	Drives EGCs formation in pSjD-TIN kidneys via CXCR5-mediated lymphocyte homing and infiltration.	(5, 19–21)
Epigenetic regulations	DNA hypomethylation	Derepresses gene expression, leading to overexpression. Hypomethylation of ISGs upregulates their expression. Specifically in B cells: DNA hypomethylation is more pronounced than in T cells and shows significant overlap with pSjD genetic risk loci.	(22, 23)
	Histone modification	Risk variants enrich in active histone marks of immune cells, upregulating pro-inflammatory genes. Histone H3 citrullination drives NETosis; NETs directly damage tubules or release antigens to form immune complexes.	(24–27)
	miRNA-155	Upregulated in pSjD, miRNA-155 targets TRF1/SOCS1/ARRB2 to activate NF-κB/JAK-STAT signaling and, upon exosomal delivery to kidneys, directly induces renal tubular epithelial cell apoptosis, inflammation, and fibrosis.	(15, 28–31)
	miRNA-146a	miR-146a is upregulated in pSjD patient PBMCs and salivary glands, where it inhibits the NF-κB pathway by targeting IRAK1 and TRAF6. Its elevated expression is associated with a higher incidence of renal disease and correlates with clinical disease activity.	(32–36)
Immune Regulation/Cytokines	DC/SGEC/RTEC	APC-mediated activation of the immune response in pSjD.	(37–39)
	IFN-I	IFN-I initiates a self-amplifying immune loop and interferon signature, which can target the kidneys, leading to lymphocytic infiltration-driven tubulointerstitial nephritis and immune complex-mediated GN.	(4)
	Macrophage	M1 and M2 macrophages, along with HIF-1α, are upregulated in pSjD TIN with hypokalemia and are involved in renal tubular injury and repair.	(40)
	B lymphocytes	Core cell in pSjD renal humoral immunity and injury. Mechanisms: 1) Autoantibody/immune complex-mediated GN; 2) Lymphocytic infiltration causing TIN.	(4)
	Autoantibodies	Key autoantibodies include systemic types (e.g., anti-SSA/SSB) linked to disease activity and renal-specific types (e.g., anti-CA II, anti-NCC) that bind tubular antigens; both can form immune complexes whose glomerular deposition activates complement and causes injury.	(41, 42, 44, 123)
	BAFF/APRIL	Critical factors for B-cell survival and activation. Serum levels are elevated in pSjD and correlate with anti-SSA/SSB/RF antibodies. Promote B-cell proliferation, plasma cell differentiation, and EGC formation.	(45–47)
	Immune complexes	Circulating or *in-situ* formed immune complexes deposit in glomeruli, activating complement and recruiting inflammatory cells, which triggers GN.	(4, 48)
	Tfh cells	Tfh cells promote B-cell responses via IL-21 and mediate follicular homing. Tfh cells and IL-21 are elevated in pSjD blood and salivary glands, and contribute to CXCR5^+^ EGC formation in target tissues.	(19, 20, 49–51, 145)
	Th1 cells	Th1 cells secrete IFN-γ, causing direct tissue damage and driving renal inflammation and fibrosis in pSjD, and are linked to severe proliferative and crescentic glomerulonephritis.	(52, 53)
	Th17 cells	Drives renal interstitial EGC formation in pSjD-TIN; its secretion of IL-17 impairs proximal tubule reabsorption by damaging Megalin/Cubilin receptors.	(5, 54)
	CD8^+^ cytotoxic T cells	In pSjD-TIN, CD8^+^ T lymphocytes are observed to directly invade renal tubular epithelial cells and induce their apoptosis.	(55)
Environmental factors	EBV	EBV triggers autoimmunity via molecular mimicry such as EBNA-1 and Ro52/60 cross-reactivity against kidneys, and through TLR activation. Cytoplasmic tubular inclusions are found in pSjD-TIN renal biopsies.	(56–59)
Endocrine factors	X chromosome inactivation escape	TLR7 on the X chromosome escapes inactivation in females with pSjD, leading to higher expression and heightened immune sensitivity; correspondingly, female NOD.B10 mice exhibit more severe kidney pathology, which is reduced upon TLR7 knockout.	(60–63)
	Estrogen/Androgens	Estrogen upregulates TLR7, forming an IFN-I positive feedback loop, while androgens protect epithelia and suppress TLR/immune activation, with menopause or local androgen deficiency elevating baseline TLR in pDCs of female pSjD patients.	(63, 64)

## 2 Genetic susceptibility

The primary evidence supporting the role of genetic factors in pSjD initially came from epidemiological data, including familial clustering, significantly higher concordance rates in monozygotic twins compared to dizygotic twins, and consistently higher prevalence of autoimmune diseases among first-degree relatives of pSjD patients (65–67). Studies on the genetic background of pSjD have been conducted using genome-wide association studies (GWAS) in populations (68). Notably, this genetic risk profile is associated not only with the overall risk of developing pSjD but also with susceptibility to key organ involvement, such as renal involvement, as well as specific clinical phenotypes.

### 2.1 Genetics

#### 2.1.1 HLA

The strongest genetic association with pSjD is found within the Human Leukocyte Antigen (HLA) region, with risk variants differing based on serological status and ethnic group (15). The HLA genes are polygenic and highly polymorphic, located on the short arm of human chromosome 6.

HLA class II genes are highly expressed in antigen-presenting cells (APCs), presenting antigenic peptides to CD4^+^ T cells and triggering immune activation. These include the HLA-DR, HLA-DQ, and HLA-DP genes (69). Consequently, these HLA class II genes are central components of autoimmune susceptibility, including in pSjD. A meta-analysis summarized high-risk loci for pSjD within the HLA class II genes, including HLA-DQA1*05:01, DQB1*02:01, and DRB1*03:01. Among these, HLA-DRB1*03:01 has been established as one of the strongest genetic risk factors for pSjD, indicating that genetic variation in HLA class II molecules confers pSjD a common susceptibility background shared with other autoimmune diseases (7, 15).

Gottenberg et al. identified a significant association between the HLA-DRB1*03:01 allele and the presence of anti-SSA and anti-SSB antibodies in patients. Positive anti-SSB and anti-SSA antibodies are risk factors for renal involvement. This suggests that HLA class II molecules may impact the disease classification and severity of pSjD by regulating autoantibodies (8, 9, 70). According to epitope binding predictions, 24 peptides derived from SSB, SSA, alpha fodrin (AF), and the muscarinic acetylcholine receptor M3 (mAChR-M3) display structural complementarity with the antigen-binding groove encoded by the HLA-DRB1*03:01 allele. These peptides are expected to form high-affinity complexes with the HLA-DRB1*03:01 molecule. Consequently, this preferential antigen presentation may drive excessive activation of CD4^+^ T cells in individuals carrying this risk allele (7). Moreover, the ancestral haplotype 8.1 (AH8.1), which includes HLA-DRB1*03:01, has been linked to persistent immune dysregulation, marked by overactive B cells and elevated proinflammatory cytokines (10). These immune abnormalities contribute to T and B cell dysregulation, which may promote renal involvement in pSjD patients. Therefore, the HLA-DRB1*03:01 allele may represent one of the genetic factors contributing to renal damage in pSjD patients.

However, the pathological manifestations of renal involvement in pSjD are diverse, and the genetic background may differ depending on the pathological subtype. Multiple studies have found that primary Sjögren’s disease-associated membranous nephropathy (pSjD-MN) and idiopathic membranous nephropathy (iMN) share the pathogenic characteristics mediated by phospholipase A2 receptor (PLA2R) autoantibodies (11, 12). In typical iMN, this specific autoantibody has been shown to be independently strongly associated with the HLA-DRB1*15:01 allele. This indicates that the PLA2R-positive glomerular lesion in pSjD-MN may have an immunogenetic background similar to that of iMN, suggesting that the glomerular lesion may involve an immunogenetic mechanism different from the interstitial nephritis observed in classical pSjD (13). Although HLA class II alleles are associated with pSjD and its renal involvement, the precise mechanisms by which different HLA class II genes induce renal damage are still unclear. It is currently proposed that differences in the biochemical characteristics of amino acids at key sites where various HLA class II molecules bind during antigen peptide presentation to T cells may influence the repertoire of autoantigenic peptides. This, in turn, could promote T cell activation and breach immune tolerance, potentially providing clues about the involvement of different HLA class II genes in the pathogenesis (71).

The HLA class III region produces molecules essential for controlling inflammation, stress responses, and innate immune functions, including major mediators like tumor necrosis factor (TNF), complement factors, and heat shock proteins (72). In European cohorts with pSjD, the TNF-2 variant at position -308 of the TNF-α gene within the HLA class III region is found at a notably elevated frequency, marking it as a significant genetic risk factor for the disease (14). The TNF-α produced by this allele is expressed in the duct cells of the minor salivary glands in patients with pSjD. This cytokine promotes the infiltration of monocytes into the salivary glands. Furthermore, it facilitates the migration of these monocytes to distant sites such as the renal epithelium, where they participate in the pathogenesis of tubulointerstitial nephritis (73, 74).

In pSjD patients, those exhibiting proteinuria or distal renal tubular acidosis show a higher frequency of the TNF-2 allele than patients without these renal manifestations. This correlation directly implicates the allele in the process of kidney injury in pSjD. From a serological perspective, individuals carrying the TNF-2 allele often exhibit heightened systemic immune responses, reflected by increased IgG levels and elevated titers of anti-SSA/SSB antibodies. Importantly, the TNF-2 allele exhibits significant linkage disequilibrium with the HLA-DR3 gene located on the HLA class II locus, suggesting that HLA class II and III genes synergistically contribute to the pathogenesis of pSjD and its renal involvement (14). HLA class II genes primarily shape the specificity of adaptive immune responses, while HLA class III genes support a wider pro-inflammatory environment.

However, most current genetic studies are based on European populations, with limited data from Asian populations. Consequently, the specific cellular and molecular pathways through which HLA alleles cause renal damage in pSjD remain unclear and must be elucidated through further functional experimentation.

#### 2.1.2 IRF5/STAT4

Early genetic studies on pSjD primarily focused on candidate genes previously implicated in immune function or other autoimmune diseases. In addition to significant associations with the HLA region, several non-HLA genes have also been shown to be associated with pSjD susceptibility. A large-scale pSjD genetic study indicated that risk alleles at loci for IRF5, STAT4, IL12A, BLK, CXCR5, and TNIP1 also show strong genetic susceptibility to pSjD.

IRF5 and STAT4 genes are the most significant non-HLA susceptibility genes in patients with pSjD (68). The IRF5 gene is located on chromosome 7q32.1 and encodes the transcription factor Interferon Regulatory Factor 5 (IRF5). It mediates the Toll-like receptor (TLR)-MyD88 signaling pathway to induce the expression of inflammatory genes and downstream interferon-related genes, thereby activating the type I interferon (IFN-I) system (75, 76). The STAT4 gene is located on chromosome 2q32.2-32.3 and encodes Signal Transducer and Activator of Transcription 4 (STAT4). It regulates the transcription of IFN-related genes and works synergistically with IRF5 to participate in IFN-I system activation (77, 78). This genetic predisposition results in sustained high expression of interferon-stimulated genes (ISGs) in immune and glandular cells, establishing a pro-inflammatory environment that favors excessive T and B cell responses. This renders the immune system in pSjD patients highly responsive to inflammatory signals, promoting subsequent abnormal activation of adaptive immunity (16).

Studies have shown that the overactivated IFN-I system is present in nearly every aspect of both glandular and extraglandular pathogenesis in pSjD patients (79). François et al. further explored the potential immunostimulatory role of IFN-I pathway dysregulation in renal damage in pSjD patients. This involves recruiting and activating lymphocyte infiltration into target organs, driving autoantibody and immune complex formation, and directly acting on renal tubular epithelial cells, causing abnormal activation, injury, apoptosis, and progression to renal fibrosis (4). Furthermore, studies show significant correlations between IRF5 and STAT4 risk alleles and positive anti-SSA and anti-SSB antibodies, suggesting that genetic susceptibility may indirectly contribute to renal involvement by promoting pSjD autoimmunity and clinical disease activity through regulation of autoantibodies (15). In conclusion, IRF5 and STAT4 alleles, through their involvement in the type I interferon signaling pathway and contribution to autoantibody production, may help explain how these genetic variants contribute to kidney injury in pSjD.

#### 2.1.3 BLK/CXCR5

Persistent polyclonal B-cell activation and autoantibody production are key mechanisms underlying renal involvement in pSjD (4). The BLK and CXCR5 genes are non-HLA susceptibility loci directly linked to B-cell function in genome-wide association studies of pSjD (15).

The BLK locus on chromosome 8p23.1 encodes B lymphocyte kinase (BLK), involved in B-cell signaling and nuclear factor-κB (NF-κB) activation (17). pSjD patients carrying BLK risk alleles may evade self-reactive B cell clearance by reducing BLK expression, resulting in B cell overproliferation, activation, and immune dysregulation (18). These proliferating B cells differentiate into plasma cells, producing autoantibodies that form immune complexes or directly target renal tissue. Alternatively, they can infiltrate the renal tubulointerstitium, causing fibrosis and kidney damage (4).

CXCR5, located on chromosome 11q23.3, encodes the C-X-C chemokine receptor 5 (CXCR5), which is expressed on B lymphocytes and follicular helper T (Tfh) cells. Its ligand is C-X-C motif chemokine ligand 13 (CXCL13). CXCR5 and CXCL13 form the CXCR5-CXCL13 axis, mediating lymphocyte homing to lymphoid follicles in secondary lymphoid organs (19). In pSjD patients carrying the CXCR5 risk allele, CXCR5^+^ B lymphocytes are reduced in peripheral blood but accumulate in target organs such as minor salivary glands (80). This is accompanied by increased CXCL13 expression, which correlates with CXCR5^+^ lymphocyte infiltration and immune dysregulation in affected tissues (81). Notably, ectopic germinal centers (EGCs), representing the abnormal accumulation of mature lymphocytes in non-lymphoid tissues, have been observed in the minor salivary glands and renal tubulointerstitium of pSjD patients, which are usually considered manifestations of chronic inflammation (5, 82). Consistent with the immunopathological characteristics of pSjD, the formation of these ectopic lymphoid structures is mainly driven by the specific expansion and targeted infiltration of CXCR5^+^ Tfh cells (20). The pathogenic relevance of this chemokine axis has been further confirmed in experimental models: targeting CXCL13 to inhibit the CXCR5-CXCL13 signaling axis can significantly reduce local lymphocyte infiltration, effectively mitigate focal tissue damage, and preserve glandular function (81). These findings suggest that CXCR5-mediated lymphocyte homing plays a key role in orchestrating EGC formation and pSjD-related immune kidney injury.

In summary, BLK and CXCR5 risk genes play crucial roles in the genetic susceptibility to renal damage in pSjD. The BLK risk gene induces excessive production and activation of autoreactive B cells. Meanwhile, abnormal activation of the CXCR5-CXCL13 axis caused by the CXCR5 risk allele recruits hyperactivated B lymphocytes and Tfh cells to the kidney. Locally in the kidney, lymphocytes infiltrating the renal interstitium can cause interstitial nephritis, while activated B cells further differentiate into plasma cells, producing autoantibodies that directly attack the kidneys or, through antigen-antibody immune complex deposition, activate complement-mediated damage to glomeruli and tubules. This process is consistent with the pathological manifestations of renal involvement in pSjD (4).

### 2.2 Epigenetics

Epigenetics modulates gene activity without altering the DNA sequence. In pSjD, these mechanisms are implicated in both systemic immune imbalance and injury to target organs. Research has focused on several key processes, including DNA methylation, histone modifications, and non-coding RNAs. Although few large-scale studies have directly connected epigenetic markers to renal injury in pSjD, multiple reports have detected abnormal epigenetic patterns in patients, providing indirect evidence for mechanisms that may drive kidney damage.

#### 2.2.1 DNA methylation

DNA methylation can regulate gene expression through chromatin remodeling, leading to changes in the accessibility of transcription factor complexes to gene regulatory regions (83). Studies have shown that pSjD patients exhibit extensive DNA hypomethylation patterns of genetic susceptibility genes in peripheral blood monocytes and target organs, such as salivary gland cells and salivary gland epithelial cells. These include hypomethylation of genes encoding lymphotoxin-alpha (LT-α), IFN-induced genes, solute carrier family members, and genes involved in naive CD4^+^ T lymphocyte development (84). Among these genes, IFI44L and MX1 exhibit the most pronounced hypomethylation in individuals positive for anti-SSA and anti-SSB antibodies. This hypomethylation leads to marked upregulation of ISGs, contributing to the systemic interferon signature characteristic of pSjD (22, 23). Notably, Miceli et al. found that the DNA methylation pattern in B cells is more significantly dysregulated than in T cells in pSjD patients. Moreover, the hypomethylated regions in B cells show extensive overlap with genetic risk loci for pSjD, with the hypomethylation of the CXCR5 gene being significantly correlated with its upregulation, resulting in a marked increase in CXCR5 mRNA levels in pSjD patients (23). The overexpression of key risk alleles driven by DNA hypomethylation collectively breaks immune tolerance, providing an upstream mechanism for lymphocyte homing and infiltration into the kidneys.

#### 2.2.2 Histone modifications

Histone modifications can regulate gene expression through chromatin remodeling, leading to changes in the accessibility of transcription factor complexes to gene regulatory regions (85). Miceli-Richard et al. discovered that genetic risk variants associated with pSjD are significantly enriched in genomic regulatory regions with active histone marks in B lymphocytes and monocytes, such as enhancers and promoters. In these regions, abnormal histone marks lead to upregulation of immune-related genes. This promotes excessive proliferation and activation of B lymphocytes and monocytes, contributing to immune system imbalance (24).

Furthermore, citrullination of histone H3, a type of post-translational modification, acts as a distinct activating signal that facilitates chromatin depolymerization in neutrophils and promotes the creation of neutrophil extracellular traps (NETs) (25). Peng et al. found that neutrophils from patients with pSjD exhibit mitochondrial damage and produce higher levels of reactive oxygen species (ROS), which enhance NET formation and promote tissue injury. This pathway appears to be abnormally active in pSjD (26). Increasing experimental and clinical evidence indicates that NETs can damage kidneys in autoimmune diseases through several mechanisms. Proteins released from NETs, including histones and myeloperoxidase, are directly toxic to renal tubular epithelial cells. Additionally, citrullinated proteins and nucleic acids from NETs can stimulate autoantibody production and immune complex formation, which deposit in the kidney. These events further activate the complement system, recruit inflammatory cells, and drive renal fibrosis (27).

Reflecting this interplay between innate and adaptive immune dysregulation, the neutrophil-to-lymphocyte ratio (NLR) is a readily accessible index of systemic inflammation and has been proven to effectively mirror the state of systemic immune imbalance in pSjD. Clinical studies demonstrate that NLR levels are significantly elevated in pSjD patients compared to healthy controls, and the degree of elevation is independently and positively correlated with disease activity, highlighting its superior diagnostic value in assessing systemic immune imbalance (86, 87). While direct evidence regarding NLR in pSjD-associated kidney injury remains scarce, its clinical utility has been extensively validated in lupus nephritis (LN), another hallmark autoimmune renal disease. In LN, an elevated NLR positively correlates with serum creatinine, proteinuria, and renal pathological activity scores, while inversely correlating with the estimated glomerular filtration rate, serving as an independent predictor of renal involvement and poor prognosis (88–91). This association likely reflects immune tolerance defects caused by epigenetic dysregulation, particularly excessive NET formation and abnormal lymphocyte activation. Future studies should explore combining easily accessible clinical indices, such as NLR. This approach may provide a practical and reliable strategy for monitoring kidney injury in pSjD, linking systemic immune imbalance with organ-specific damage.

#### 2.2.3 miRNA

MicroRNAs (miRNAs) are short non-coding RNAs, usually 18-25 nucleotides in length, that control gene expression through interaction with the 3’untranslated regions of mRNAs (92). In pSjD, miRNAs play important roles in modulating immune responses and inflammatory processes. For example, miR-155 and miR-146a form a feedback loop regulating key inflammatory pathways, including NF-κB. This mechanism has been widely validated in autoimmune disorders (93). NF-κB, a key transcription factor for genes involved in immunity and inflammation, is central to regulating inflammatory responses. Beyond systemic autoimmune effects, NF-κB contributes to renal inflammation and fibrosis in diseases including IgA nephropathy, diabetic nephropathy, and lupus nephritis (94, 95).

miR-146a functions as a key negative feedback regulator in immune responses. It suppresses key downstream molecules in the TLR pathway, including Interleukin-1 Receptor-Associated Kinase 1 (IRAK1) and Tumor Necrosis Factor Receptor-Associated Factor 6 (TRAF6), thus modulating the NF-κB inflammatory pathway through negative feedback. Consequently, decreased levels of miR-146a can facilitate inflammation and trigger autoimmune responses (32). Interestingly, levels of miR-146a/b are markedly increased in the peripheral blood mononuclear cells (PBMCs) and salivary gland tissues of pSjD patients. However, the persistent interferon signature in pSjD indicates that this feedback mechanism may not fully function. This impairment reflects disease severity, as PBMC miR-146a/b levels show a positive correlation with visual analog scale scores for parotid swelling and dry eyes (33–35). More importantly, Jiang et al. reported that anti-SSA-positive pSjD patients with elevated miR-146a levels were more likely to develop renal involvement (36). These findings indicate that miR-146a upregulation may serve as a compensatory mechanism in pSjD, yet it is insufficient to suppress ongoing inflammation. Consequently, miR-146a levels are associated with immune imbalance, clinical symptoms, and disease progression. Overall, these observations indicate that while miR-146a upregulation may act as a compensatory mechanism in pSjD, it cannot fully counteract ongoing inflammation, resulting in immune imbalance and correlations with clinical symptoms and disease progression.

In contrast, miR-155 is a potent pro-inflammatory factor that is significantly elevated in patients with pSjD (15). Zhang et al. established a pSjD inflammation model by treating salivary gland epithelial cells with interferon-gamma (IFN-γ). They observed a significant upregulation of miR-155 in these epithelial cells, which negatively regulates β-arrestin 2 (ARRB2), thereby activating the NF-κB signaling pathway and promoting inflammation and apoptosis (28). ARRB2, a crucial signaling regulator, is also expressed in diverse tissue cells including renal tubular epithelial cells, playing a central role in regulating cell proliferation, inflammatory responses, and tissue fibrosis (29). We speculate that the upregulated miR-155 in pSjD patients may also promote renal cell injury and apoptosis by targeting and suppressing ARRB2, thereby activating the NF-κB pathway.

Recent studies suggest that miR-155 can be delivered to renal tubular epithelial cells via exosomes. By targeting the telomere-associated protein Telomeric Repeat-binding Factor 1 (TRF1), miR-155 induces apoptosis and promotes renal interstitial fibrosis. Simultaneously, miR-155 can target and inhibit Suppressor of Cytokine Signaling 1 (SOCS1) in renal intrinsic cells, leading to abnormal activation of the Janus kinase-signal transducer and activator of transcription (JAK-STAT) pathway and enhanced pro-inflammatory responses (30, 31). These findings support that upregulated miR-155 contributes to renal injury, inflammation, and fibrosis in pSjD through multiple molecular targets.

Based on current findings, microRNAs may be involved in the development of renal injury in pSjD. Most evidence so far has focused on systemic immune dysregulation in pSjD, whereas kidney-specific mechanisms remain underexplored. Future studies should place greater emphasis on defining miRNA expression patterns and their downstream targets specifically associated with renal injury in pSjD. Such analyses could be performed using renal tissue or urinary exosomes obtained from pSjD patients, as well as kidney-specific cellular models. These approaches would help clarify how miRNAs influence the local renal immune microenvironment and contribute to fibrotic progression. Ultimately, this study may support the development of non-invasive biomarkers and targeted therapeutic strategies for renal involvement in pSjD.

## 3 Immune dysregulation

**Figure 1:**
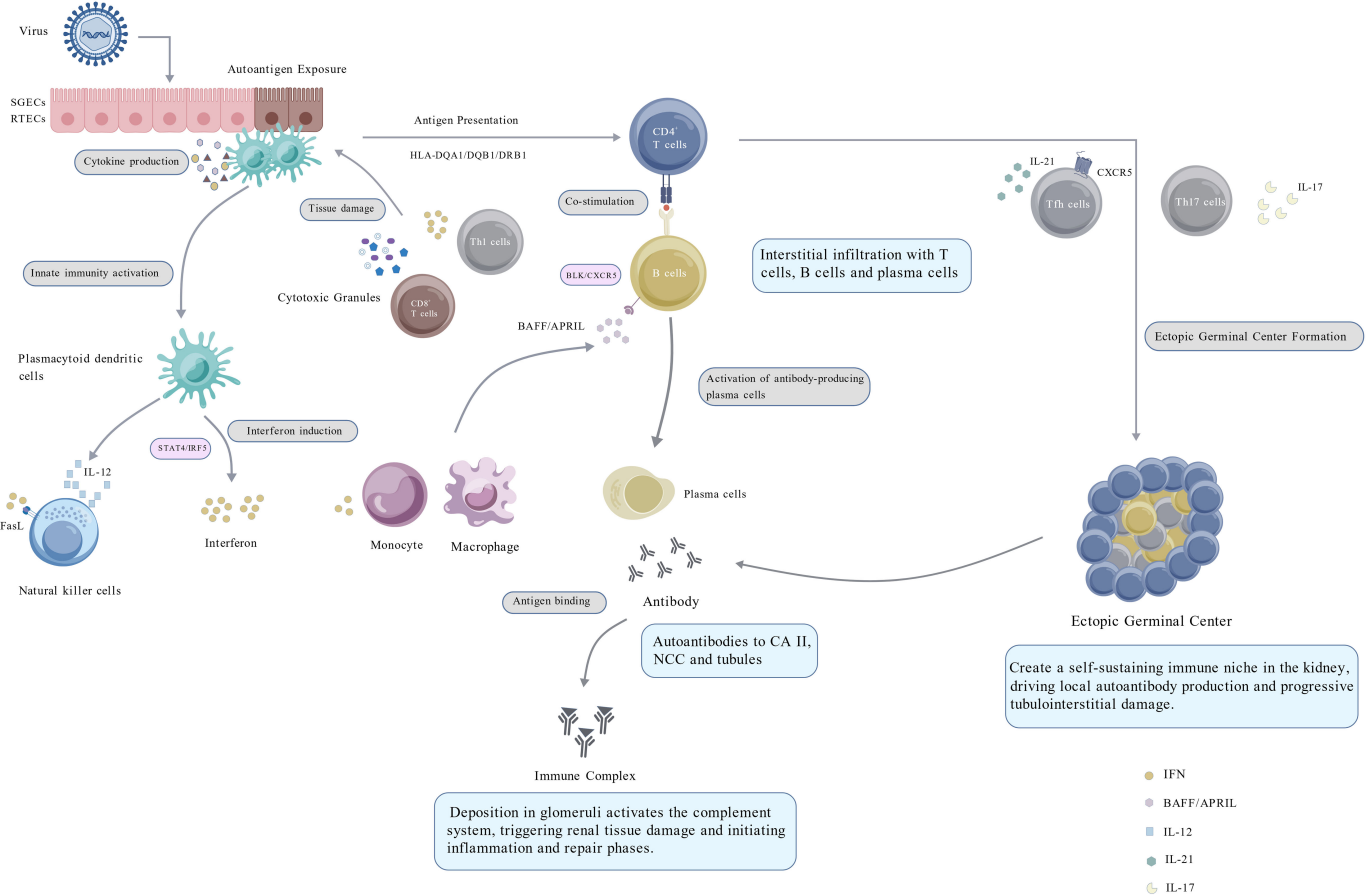
Possible pathogenic mechanisms of renal involvement in pSjD. Viral infection triggers the activation of target organ epithelial cells (SGECs/RTECs) in genetically susceptible individuals. These activated epithelial cells can function as APCs, producing a cytokine milieu of IL-6, IFNs, BAFF, and APRIL. This environment recruits immune cells, promotes pDC overproduction of IFN-I, and enhances B-cell differentiation and survival. Key cellular players include IL-21-secreting Tfh cells, which drive EGCs formation, and CD8^+^ T cells that directly kill renal tubular epithelial cells. Monocytes and macrophages also contribute significantly. These processes converge on the kidney via two primary pathways: Glomerular Injury: Immune complex deposition activates complement, causing mesangial proliferation and extracellular matrix synthesis, manifesting as MPGN. Tubulointerstitial Injury: Tubular-specific autoantibodies cause electrolyte disturbances and acidosis. Infiltration by T cells, B cells, and plasma cells leads to tubulointerstitial nephritis, exacerbated by local autoantigen expression. Renal EGCs further promote autoreactive B-cell activation and high-affinity autoantibody production. APC, antigen-presenting cell; pSjD, primary Sjögren’s disease; SGECs, salivary gland epithelial cells; RTECs, renal tubular epithelial cells; IFN, interferon; ILs, interleukins; BAFF, B cell activating factor; APRIL, A proliferation-inducing ligand; Tfh, T follicular helper; CXCR5, C-X-C motif chemokine receptor 5; EGCs, ectopic germinal centers; pDC, plasmacytoid dendritic cell; CA II, carbonic anhydrase II; NCC, sodium-chloride cotransporter; FasL, Fas ligand; MPGN, membranoproliferative glomerulonephritis; STAT4, signal transducer and activator of transcription 4; IRF5, interferon regulatory factor 5; BLK, B lymphoid tyrosine kinase; HLA, human leukocyte antigen. Created with BioGDP.com (96).

Kidney injury in pSjD arises from the interaction between innate and adaptive immune responses, which gradually leads to autoimmune activation and loss of immune tolerance. Aberrant activation of the innate immune system, largely driven by type I interferon (IFN-I) signaling, precedes and promotes maladaptive adaptive immune responses. As these immune pathways remain active, immune cells and effector molecules accumulate within renal tissue and sustain a localized inflammatory milieu. This persistent inflammatory state contributes to the development of both tubulointerstitial nephritis and glomerulonephritis (**Figure 1** summarizes the possible pathogenic mechanisms of renal involvement in pSjD).

### 3.1 Innate immunity

#### 3.1.1 Innate immunity cells

Dendritic cells (DCs) include follicular dendritic cells (FDCs) and plasmacytoid dendritic cells (pDCs). Both types act as APCs, quickly detecting signals of tissue damage. They are known to activate adaptive immunity and stimulate antibody production. Studies have found that both FDCs and pDCs are increased in the salivary glands of pSjD patients. This suggests that DCs play an important role in the early immune response of the disease (37). DCs recognize danger signals through Toll-like receptors (TLRs) and respond by releasing IFN-I and other pro-inflammatory cytokines, thereby activating the immune system. In individuals genetically prone to pSjD, salivary gland epithelial cells (SGECs) can also act as non-professional APCs. They do this by increasing MHC class II expression, which helps trigger immune responses (38). IFN-α secreted by SGECs directly contributes to local tissue injury in the salivary glands. Nucleic acids released from dying SGECs can stimulate pDCs through TLR7 or TLR9, further promoting inflammation (64, 97). Notably, renal tubular epithelial cells (RTECs) have similar structural and functional features with SGECs. Under inflammatory conditions, RTECs can upregulate MHC class II molecules and costimulatory molecules such as B7-1 (CD80) and B7-2 (CD86), thereby acquiring the capacity to present antigens to T cells (98). In pSjD patients with tubulointerstitial nephritis, tubular epithelial cells have been shown to express costimulatory molecules, suggesting that local antigen presentation within the kidney may contribute to ongoing tissue injury and the formation of ectopic germinal centers (39).

Macrophages, key effector cells of innate immunity, have recently gained attention for their role in pSjD kidney damage. Analyses of kidney tissues from pSjD patients with TIN reveal prominent infiltration by macrophages. Additional studies indicate that in renal tissues from pSjD patients with hypokalemia, the expression levels of hypoxia-inducible factor 1-alpha (HIF-1α), M2 macrophages and M1 macrophages were all elevated. Under normal conditions, M2 macrophages and HIF-1α are seldom detected in the renal tubulointerstitial area. These observations suggest that M2 macrophages may contribute to acute tubular injury and help regulate subsequent repair in pSjD (40). Future research should aim to define the specific functions of different macrophage subsets in pSjD kidney damage and assess their potential as therapeutic targets.

#### 3.1.2 IFN-I

IFN-I, a major cytokine released when pDCs are activated, can stimulate pDCs through both autocrine and paracrine signaling, creating a persistent feedback loop that amplifies their activation (99). IFN-I also upregulates TLR7 on B lymphocytes via the JAK-STAT pathway, enhancing their sensitivity to endogenous nucleic acids and promoting IFN-α production within these cells. This forms an additional positive feedback mechanism, which further intensifies autoimmune activity (100). The type I interferon system includes IFN-α and IFN-β, with their receptors broadly present on almost all nucleated cells. Binding of IFN-I to its receptor activates the downstream JAK-STAT pathway, which drives the transcription and expression of ISGs. Proteins encoded by ISGs participate in various physiological and pathological processes, including host defense against microbes and tumors. They act as key mediators connecting innate and adaptive immunity (101). The upregulation of ISGs by IFN-I is referred to as the type I interferon signature, a hallmark of immune dysregulation in pSjD that contributes to disease activity and organ involvement (102).

In a UK cohort, Bodewes et al. identified an association between systemic IFN system activation and elevated lung and kidney disease activity scores on the European League Against Rheumatism Sjögren’s Syndrome Disease Activity Index (ESSDAI) in pSjD patients (103). Evidence indicates that IFN-I signaling stimulates intrinsic renal cells to secrete cytokines. These cytokines recruit inflammatory infiltrates, driving renal injury and cell death (104). In IL-14α pSjD mice, renal lymphocyte infiltration was more prominent compared to mice lacking IFN-I receptors, indicating the crucial role of IFN-I signaling in kidney injury associated with pSjD (105). While earlier studies emphasized IFN-I production by pDCs in kidney disease, recent evidence suggests that intrinsic renal cells may also produce IFN-I (106). Cytoplasmic double-stranded RNA can activate the IFN-I pathway via TLR3 on podocytes, mesangial cells, endothelial cells, and tubular epithelial cells, resulting in localized kidney inflammation (107).

IFN-I is a key cytokine that drives kidney injury in pSjD, rather than serving only as a background inflammatory signal. Together with T and B lymphocytes, it forms a self-amplifying loop that promotes renal damage. Under genetic susceptibility and environmental triggers, DCs, SGECs and RTECs produce IFN-I. This cytokine binds to receptors on lymphocytes, activating T and B cells through multiple signaling pathways (100). By upregulating cyclin D2 and the anti-apoptotic protein BCL-2, IFN-I enhances B cell proliferation and survival (108–110). It strengthens B cell responses in germinal centers and promotes extrafollicular B cell differentiation into autoantibody-secreting plasma cells (111, 112). IFN-I can also further drive B cell activation and promote immunoglobulin class switching by upregulating B-cell activating factor (BAFF) and a proliferation-inducing ligand (APRIL) expression (16). Through the stimulator of interferon genes (STING) pathway, it induces B cells to express CXCR5 and release IL-6, contributing to abnormal activation of Tfh cells (113). In line with this, recent evidence suggests that IFN-I can induce the expression of CXCL13 in tissue-resident fibroblasts, drive the recruitment of CXCR5-dependent B cells, and promote the formation of ectopic germinal centers in non-lymphoid tissues (114). In pSjD, the CXCL13/CXCR5 chemokine axis plays a central role in recruiting CXCR5-positive B cells and coordinating the formation of EGC-like structures in target organs, which has been confirmed by multiple studies (115, 116). In pSjD patients with TIN, EGCs containing active B cells and Tfh cells also exist in the kidney, indicating that lymphocytes maintain local autoimmune activity and antibody production, and drive continuous renal damage (5).

IFN-I further drives CD4^+^ T cell differentiation toward Th1, activates memory CD8^+^ T cells, and induces Fas ligand expression in natural killer (NK) cells, leading to apoptosis of target cells. Additionally, IFN-I induces chemokines CXCL9, CXCL10, and CXCL11, which attract CXCR3-positive pDCs and lymphocytes (117–119). Autoantibodies secreted by plasma cells can combine with nucleic acids from dying cells to form immune complexes. These complexes are internalized by pDCs, triggering TLR7/9 signaling and inducing further IFN-α production, completing a self-reinforcing pathogenic cycle (120).

Overall, in an IFN-I-dominated environment, adaptive immunity is fully activated, immune tolerance is lost, and multiple pathogenic loops collectively drive kidney damage in pSjD.

### 3.2 Adaptive immunity

#### 3.2.1 B lymphocyte

Perinephric lymphocyte infiltration and pronounced B cell activation are central contributors to kidney damage in pSjD patients (121). Importantly, B lymphocytes not only drive autoantibody production but also participate in disease pathogenesis via immune complex deposition and increased expression of BAFF.

##### 3.2.1.1 Autoantibodies

Autoantibodies are major contributors to kidney damage in patients with pSjD. Elevated serum autoantibody titers, predominantly anti-SSA/Ro52 and anti-SSA/Ro60, provide strong evidence for antibody-mediated pathology in pSjD (41). Jasiek et al. demonstrated that the presence of anti-SSA, anti-SSB, and rheumatoid factor (RF), particularly at high titers, correlates with an unfavorable renal prognosis in pSjD (42). A case report from Finland identified antibodies reactive against renal tubules and glomeruli in pSjD patients with biopsy-proven TIN (122). In Ro-60 antigen-deficient mice, autoantibody-mediated immune complex deposition reportedly triggers MPGN (43).

Beyond systemic autoantibodies, antibodies targeting kidney-specific antigens act as direct effector molecules in pSjD-related renal injury. Multiple studies have confirmed the presence of anti-carbonic anhydrase II (CA-II) antibodies in pSjD patients with renal involvement (123–125). CA-II is a key enzyme that regulates acid-base homeostasis in erythrocytes, aqueous humor, and renal tubules by catalyzing the reversible hydration of carbon dioxide (126). Takemoto et al. reported elevated anti-CA-II levels in pSjD patients, with the highest titers observed in those presenting with distal renal tubular acidosis (dRTA) (127). Studies have found that mice immunized with CA-II develop systemic exocrine gland inflammation similar to the clinical manifestations of human pSjD. These mice also exhibit renal damage, primarily characterized by mild TIN. CA-II antibody-positive mice exhibit dRTA in ammonium chloride loading tests (128, 129). These findings indicate that elevated levels of CA-II antibodies in plasma can impair CA-II function, contributing to dRTA in certain pSjD patients. In addition, circulating autoantibodies targeting the sodium-chloride cotransporter (NCC) have been detected in the serum of pSjD patients presenting with Gitelman syndrome. Since NCC is the key transporter involved in Gitelman syndrome, this observation further supports the involvement of plasma cell-derived autoantibodies in renal injury (44). Taken together, these studies highlight the critical role of plasma cell-derived autoantibodies in mediating renal injury in pSjD.

##### 3.2.1.2 Immune complexes

In pSjD patients, large antigen-antibody complexes (ICs) often accumulate because the body cannot efficiently clear them. When these ICs deposit in the kidneys, they activate the complement system, leading to renal tissue injury and initiating inflammatory and reparative processes. This cascade constitutes a primary mechanism underlying glomerular injury in pSjD (4). Among glomerular lesions in pSjD, the most frequently observed form of glomerulonephritis is MPGN, which is secondary to cryoglobulinemia resulting from polyclonal B-cell activation. This subtype of MPGN is driven by ICs, comprising immunoglobulins (IgG, IgM, IgA), κ and λ light chains, and complement proteins such as C3 and C1q. These molecules can deposit in the glomeruli alone or in combination with rheumatoid factor (RF), forming cryoglobulins. Activation of the classical complement pathway generates chemotactic signals such as C3a and C5a, which recruit macrophages and neutrophils to release proinflammatory cytokines. Simultaneously, formation of the membrane attack complex (MAC) damages mesangial and subendothelial areas, stimulating mesangial cell proliferation and extracellular matrix production, and producing the characteristic double-contour appearance (48). In addition, membranous nephropathy is occasionally observed among pSjD-related glomerular lesions. Kidney biopsies often show polyclonal IgG and C3 deposits along the glomerular capillary basement membrane, indicating the involvement of both locally formed and circulating ICs (130).

##### 3.2.1.3 BAFF/APRIL

BAFF and APRIL are members of the tumor necrosis factor ligand superfamily and play central roles in maintaining peripheral B-cell homeostasis. Their biological functions are mainly associated with enhanced B-cell proliferation and prolonged survival. By binding to the BAFF receptor (BAFF-R), BAFF drives the maturation of immature B cells. In addition, BAFF supports plasma cell survival and immunoglobulin secretion through signaling via the B-cell maturation antigen (BCMA). In mature B cells, BAFF further modulates differentiation programs through interaction with the transmembrane activator and CAML-interacting protein (TACI). In contrast, APRIL mediates its immunological effects primarily through BCMA and TACI (131).

A characteristic feature of immune imbalance in pSjD is the dysregulation of signaling mediated by BAFF and APRIL. Previous studies have reported significantly higher serum BAFF and APRIL levels in pSjD. These increases show positive associations with anti-SSA, anti-SSB, and RF titers (45, 46). Such observations support the presence of a systemic immune milieu favoring persistent autoantibody production and support a mechanistic basis for immune-mediated renal injury. Immunohistochemical analyses demonstrate that SGECs are capable of producing BAFF. Meanwhile, infiltrating BAFF-positive monocytes and T lymphocytes, together with BAFF-R-expressing B cells, are commonly detected within affected glands. Notably, this local immune architecture closely resembles the BAFF/APRIL-enriched cellular infiltration observed in renal tissues from lupus nephritis and idiopathic membranous nephropathy (47, 132, 133).

Experimental studies further support a pathogenic role for excessive BAFF signaling. In pSjD mouse models, BAFF overexpression enhances lymphocyte infiltration and EGC formation in salivary gland tissues (47). Comparable lymphoid aggregates with EGC features have also been identified in renal biopsy specimens from pSjD patients with kidney involvement (5). Related observations in lupus nephritis provide additional mechanistic insight that may be relevant to pSjD. Dorraji et al. demonstrated that tubulointerstitial cells in lupus nephritis express chemokines and adhesion molecules that facilitate lymphocyte recruitment and promote the organization of EGCs. Furthermore, elevated renal BAFF expression in lupus mice promotes plasma cell infiltration within these EGCs (134, 135). Crucially, both the frequency and magnitude of serum BAFF elevation are greater in pSjD than in systemic lupus erythematosus (136). This suggests that BAFF upregulation may also occur within the kidneys of pSjD patients. Such upregulation may support intrarenal lymphocyte and plasma cell infiltration, EGC formation, and subsequent progression toward renal fibrosis. An interventional study is consistent with this interpretation. In pSjD animal models, pharmacological blockade of the BAFF pathway using low-molecular-weight compounds leads to a reduction in autoantibody levels. Such treatment also limits B-cell infiltration in both lacrimal glands and renal tissues (137). These interventional data strongly suggest that targeting BAFF could be a viable therapeutic strategy.

Taken together, available evidence indicates that the BAFF/APRIL axis contributes substantially to pSjD-associated renal injury. This contribution is mediated through sustained aberrant B-cell activation, immune cell accumulation, and EGC development. In addition, elevated IFN-I levels in pSjD may further amplify BAFF production by monocytes, thereby reinforcing prolonged polyclonal B-cell activation (138). Future studies directly examining BAFF expression in pSjD kidney biopsies will be crucial to validate this pathogenic model.

#### 3.2.2 T lymphocyte

Antigen presentation is essential for initiating adaptive T-cell responses and plays a pivotal role in the renal immune pathology observed in pSjD. This process contributes to disease development by promoting proinflammatory cytokine release and triggering B-cell activation. A multicenter French pathological study analyzing 95 renal biopsies from pSjD patients with tubulointerstitial nephritis revealed extensive infiltration by T lymphocytes, B lymphocytes, and plasma cells, with CD4^+^ T cells predominating (42, 55, 139, 140). Inflamed glandular tissues in pSjD contain diverse subsets of CD4^+^ effector T cells, including Th1 cells producing IFN-γ, Th17 cells producing IL-17, and Tfh cells producing IL-21. Notably, the lymphocytic infiltrates in the renal tubulointerstitium of pSjD patients mirror those observed in salivary gland epithelia, suggesting a conserved mechanism of tissue pathology (42). These patterns highlight the critical role of T-cell-mediated immunity in renal lesions of pSjD and indicate that distinct T-cell subsets may contribute to renal injury through complementary inflammatory, helper, and cytotoxic mechanisms.

##### 3.2.2.1 Th1 cells

Th1 cells are the main CD4^+^ T-cell subset responsible for cellular immune injury in the kidneys of pSjD patients. Their levels are elevated in both serum and salivary gland tissues. When exposed to IL-12, Th1 cells produce IFN-γ, which contributes to tissue inflammation and damage (141). Although direct evidence in human pSjD kidneys remains limited, mechanistic insights from animal models of autoimmune nephropathy provide supporting evidence for a potential pathogenic role of Th1 cells in renal inflammation. In autoimmune nephropathy models, IL-12-stimulated Th1 cells secrete IFN-γ, which recruits macrophages and CD8^+^ T cells into glomeruli and the tubulointerstitial compartment. This recruitment can promote proliferative and crescentic nephritis and lead to interstitial fibrosis (52). Dorraji et al. showed that mice lacking the Th1-associated transcription factor T-bet had lower proteinuria, fewer glomerular crescents, and less tubulointerstitial inflammation compared with wild-type C57BL/6 mice (53). These results highlight the importance of Th1 cells in mediating renal injury. In addition to their direct pro-inflammatory effects, Th1-derived IFN-γ can stimulate monocytes to secrete BAFF and increase BAFF receptor expression on RTECs. This creates a local environment that supports abnormal B-cell proliferation and autoantibody production (142, 143).

Taken together, these observations suggest that Th1 cells may contribute to renal damage in pSjD both by driving inflammation through IFN-γ secretion and by promoting B-cell activation via local BAFF induction. Further studies are needed to clarify the detailed molecular mechanisms underlying these processes.

##### 3.2.2.2 Th17 cells

Katsifis et al. demonstrated that key cytokines required for the proliferation of Th17 cells, a subset of CD4^+^ lymphocytes, such as IL-6 and IL-23, as well as the primary pro-inflammatory cytokine IL-17 secreted by Th17 cells, are significantly elevated in the serum and salivary gland tissues of pSjD patients, with IL-17 levels correlating with the severity of histopathological damage (54). In a pSjD mouse model, IL-17 gene knockout was shown to prevent disease onset (144). Wang et al. identified EGC formation in the renal interstitium of pSjD patients presenting with proximal renal tubular acidosis (pRTA). Furthermore, IL-17 secreted by Th17 cells is significantly increased and diffusely distributed within the proximal tubule region, coinciding with the downregulation of Megalin and Cubilin receptors expressed on the apical membranes of proximal tubular epithelial cells. Notably, IL-17 levels correlate negatively with Megalin expression, both of which are closely associated with EGC formation. These findings suggest that Th17 cell infiltration and EGC formation may mediate renal-specific proximal tubular dysfunction in pSjD by downregulating Megalin and Cubilin receptors, thereby linking local immune activation to functional tubular injury (5).

##### 3.2.2.3 Tfh cells

Together with BAFF/APRIL signaling, Tfh cells provide critical helper signals that sustain intrarenal B-cell activation and ectopic germinal center maintenance in pSjD. Studies from multiple independent pSjD cohorts have described increased frequencies of activated Tfh cells, along with elevated IL-21 levels, in peripheral blood and salivary gland tissues when compared with healthy controls. In parallel, these studies noted that higher proportions of activated Tfh cells tended to associate with increased ESSDAI scores, suggesting a relationship between Tfh cell levels and disease activity in pSjD patients (49–51, 145). A defining feature of Tfh cells is their high expression of the chemokine receptor CXCR5, which facilitates their recruitment to secondary lymphoid structures. Within such environments, IL-21 production and co-stimulatory interactions contribute to B-cell maintenance and activation, thereby favoring the differentiation of B cells into antibody-secreting plasma cells (146). Multiple pathological biopsies have demonstrated that the renal interstitium of patients with pSjD and concurrent TIN exhibits dense inflammatory infiltration dominated by CD4^+^ T cells, with structurally intact EGCs also evident within the renal parenchyma (5, 42, 147). Notably, in the salivary glands, the classic target organ of pSjD, CXCR5^+^ Tfh cells with high IL-21 expression are well established as key drivers of ectopic lymphoid structure formation (20). In other forms of chronic kidney disease, Tfh cells similarly contribute directly to the development of tertiary lymphoid structures and renal fibrosis in an IL-21 dependent manner (21). These observations suggest that Tfh cells governed by the CXCR5/IL-21 axis very likely infiltrate the kidneys in pSjD associated TIN as well, perpetuating sustained immune mediated injury through the promotion of EGC formation and the proliferation and activation of B cells.

Besides CD4^+^ T-cell subsets, evidence also points to a more direct involvement of CD8^+^ cytotoxic T lymphocytes in renal tissue injury in pSjD. Matsumura et al. observed that CD8^+^ T cells are able to infiltrate RTECs and release cytotoxic granules, leading to epithelial apoptosis and tubular inflammatory damage (55).

Overall, renal injury in pSjD appears to involve the coordinated effects of multiple T-cell populations rather than a single dominant subset. In this context, Tfh-related B-cell activation, Th1/Th17-associated inflammatory responses, and CD8^+^ T-cell–mediated cytotoxic effects all contribute to the pathological features of pSjD-associated tubulointerstitial nephritis, including lymphocytic infiltration, ectopic germinal center formation, and tubular epithelial injury.

## 4 Environmental factors

Environmental factors are key triggers of innate immune activation in individuals with genetic susceptibility (4). Among these, viral infections are considered major environmental contributors in pSjD, potentially initiating sustained interferon responses (148). Evidence shows that several core antiviral pathways of the innate immune system, including TLR signaling and IFN-I system, are markedly activated in pSjD patients (102). Viral nucleic acids, such as double-stranded RNA, single-stranded RNA, and unmethylated CpG DNA, can stimulate plasmacytoid dendritic cells (pDCs) through TLR3, TLR7/8, and TLR9, leading to production of IFN-I and proinflammatory cytokines. This contributes to the persistent interferon signature characteristic of pSjD (149).

Epstein-Barr virus (EBV) has been the most extensively studied pathogen in pSjD. Viral microRNAs encoded by EBV can mimic host signals and chronically activate TLR7/8 in pDCs, resulting in sustained overproduction of IFN-α. This overproduction may contribute to the long-term interferon signature observed in pSjD (150). Croia et al. reported that EBV-infected follicular plasma cells in salivary gland germinal centers can react with the autoantigen SSA/Ro52. This suggests that EBV may promote systemic autoimmunity in pSjD via molecular mimicry, potentially contributing to multi-organ involvement (56).

In systemic lupus erythematosus, EBV nuclear antigen 1 (EBNA-1) shares critical amino acid sequences with the autoantigen SSA/Ro60, allowing antibodies and T cells targeting EBNA-1 to cross-react with Ro60-expressing tissues (57). Since SSA/Ro60 is also a core autoantigen in pSjD, a similar molecular mimicry mechanism may underlie autoimmune dysregulation in this disease. Renal biopsies from pSjD patients with TIN can reveal cytoplasmic tubular inclusions, interpreted as a nonspecific response to viral injury, implying the possibility of active local viral replication in the kidney (58). Grywalska et al. found serological evidence of EBV reactivation in primary glomerulonephritis, correlating with proliferative renal lesions (59). Cross-reactive antibodies against EBNA-1 may deposit in glomeruli, causing proteinuria and nephritis-like changes, providing indirect support for a viral molecular mimicry mechanism in pSjD renal injury (151). Other viruses, such as cytomegalovirus (CMV), coxsackievirus, and human T-cell leukemia virus type I (HTLV-1), have been associated with pSjD, though their role in renal involvement remains unclear and requires further study.

## 5 Endocrine regulation

pSjD shows a strong female predominance, with an estimated male-to-female ratio of 1:9 to 1:20 (4). The exact mechanisms through which sex affects kidney injury in pSjD remain unclear, and clinical cohort studies have not yet confirmed this relationship. In a NOD.B10 pSjD mouse model, Achamaporn et al. observed that female mice showed less severe lacrimal gland inflammation but more severe renal lesions than male mice. The kidney lesions in female mice included increased lymphocyte infiltration and higher serum levels of total and reactive IgG (60). These findings indicate that both the X chromosome and sex hormones may influence the onset and progression of renal damage in pSjD.

The TLR7 gene, located on the X chromosome, has been linked to female-biased autoimmunity because it can escape X-chromosome inactivation, leading to higher expression in females than in males (61). In female pSjD patients, we observed higher TLR7 expression in the parotid and salivary glands, as well as in B lymphocytes, plasmacytoid dendritic cells, CD14^+^ monocytes, and monocyte-derived dendritic cells (62). Knocking out TLR7 in NOD.B10 female mice reduced kidney inflammation, decreased the number of pathogenic B and T cells, and lowered serum IgG levels (60). These results suggest that elevated TLR7 expression promotes broad immune activation and contributes to both glandular and renal pathology in female pSjD mice.

Estrogen Receptor Alpha (ERα) mediates the effects of estrogen and is present not only in female reproductive organs but also in most immune cells and renal tissues (152). In mouse models of immune-mediated renal injury, estrogen activates renal innate cells through ERα, alters their metabolic pathways, and triggers the release of inflammatory cytokines (153). Estrogen also increases TLR7 transcription, which further stimulates the IFN-I system and upregulates ERα expression, forming a positive feedback loop that amplifies female-biased immune responses and disrupts immune balance (63). Androgens protect epithelial cells from apoptosis, limit self-antigen release, and suppress TLR upregulation, thereby dampening immune activation. In females, lower androgen levels, for example during menopause or due to impaired local synthesis, can further increase basal TLR expression in plasmacytoid dendritic cells (64). This increase may result in hyperactivation of these cells and potential loss of immune tolerance. Overall, the interplay between X-chromosome-linked genes and estrogen signaling may underlie the female predominance observed in pSjD-related kidney injury.

## 6 Discussion

Renal involvement in pSjD arises from the interplay of genetic, immune, environmental, and endocrine factors, which together disturb immune homeostasis both systemically and within the kidneys. Understanding this complex pathogenic network is essential for clarifying disease progression and for guiding the development of targeted therapies.

Genetic factors significantly predispose individuals to renal damage in pSjD. The MHC class II molecule encoded by HLA-DRB1*03:01 exhibits high affinity for SSA and SSB peptide antigens, which determines the specificity of the autoimmune response. Variants in IRF5 and STAT4 enhance IFN-I signaling, producing a distinctive interferon signature. In parallel, BLK and CXCR5 variants are closely associated with the proliferation and activation of lymphocyte subsets. Epigenetic changes further amplify these abnormal immune responses, intensifying renal injury.

Immune dysregulation is a central factor driving kidney injury in pSjD. pDCs remain persistently active and release IFN-I, which stimulate ISGs and create a long-lasting pro-inflammatory environment in the kidney. This persistent IFN-I signaling promotes B cell activation and immunoglobulin class switching. Both BAFF/APRIL secretion and enhanced B-cell receptor (BCR) signaling contribute to this process. IFN-I also supports the proliferation and survival of CD4^^+^^ T cells, either directly or through IL-15 induction, and enhances the cytotoxic activity of CD8^+^ T cells. Renal resident cells respond to IFN-I by producing pro-inflammatory cytokines, which attract monocytes and macrophages and worsen tissue damage.

Activated B cells differentiate into plasma cells that secrete autoantibodies. Autoantibody-containing immune complexes accumulate in the glomeruli, activating complement and contributing to glomerular injury. In addition, autoantibodies targeting kidney-specific antigens can directly damage tubular epithelial cells, leading to functional impairments such as renal tubular acidosis. CD4^+^ T cell subsets act together to amplify local inflammation. Tfh cells migrate to the renal interstitium through CXCR5 and stimulate B cells via IL-21. Th1 and Th17 cells secrete IFN-γ and IL-17, recruiting inflammatory cells that aggravate tubular injury. CD8^+^ cytotoxic T cells induce tubular epithelial apoptosis through granule-mediated mechanisms. This network represents the primary immune-mediated mechanism responsible for kidney injury in pSjD.

Viral infections may breach immune tolerance in genetically susceptible individuals with pSjD. This can occur through the activation of innate immune pathways, such as TLR and IFN-I signaling. Additionally, molecular mimicry may further contribute to virus-associated renal injury. Mechanistically, the female predominance in pSjD has been linked to the escape of X-linked TLR7 from X-chromosome inactivation. Furthermore, estrogen enhances TLR7 transcription in plasmacytoid dendritic cells. These factors synergize with IFN-I signaling to amplify the autoimmune response.

Research on pSjD has increasingly aimed to connect mechanistic insights with targeted therapeutic approaches, particularly regarding kidney involvement. The JAK/STAT pathway has become a focus because inhibiting it can block downstream effects of type I interferons and several pro-inflammatory cytokines (154). Mouse models of pSjD indicate that JAK inhibitors can lessen inflammation in the salivary glands and partially restore their function (155). Clinical observations are consistent with these experimental findings. In a 2022 exploratory, non-controlled study, patients with active pSjD who received baricitinib showed significant reductions in ESSDAI scores. Among these patients, two individuals with interstitial lung disease also reported improved respiratory symptoms, and high-resolution CT scans revealed amelioration of pulmonary lesions (156). Additionally, subgroup analysis from another study indicated that pSjD patients with baseline ESSDAI ≥14 derived greater clinical benefit from JAK inhibition (157). Thus, while JAK inhibitors show promise, their efficacy in addressing renal involvement in pSjD remains unconfirmed. Prospective, randomized, and blinded trials with kidney-focused outcome measures are needed to clarify their therapeutic potential.

B lymphocytes are central to the development of pSjD, making rituximab (RTX) an important therapeutic focus. RTX can modulate T-cell responses, particularly affecting the Th17 pathway (158). Meta-analyses of RTX therapy have produced inconsistent results, leaving its overall clinical effectiveness uncertain. For example, Gottenberg et al. observed improvements in ESSDAI scores following RTX treatment, whereas studies by Devauchelle-Pensec et al. and Bowman et al. did not reproduce these outcomes (159). Recent clinical data from France showed that among six pSjD patients with renal involvement, five experienced clinical improvement after RTX therapy (160). Telitacicept is a dual-targeted inhibitor that simultaneously acts on BAFF and APRIL. In a Phase III clinical trial for pSjD, the drug met its primary endpoint, significantly reducing ESSDAI and improving immunological markers (161). In another clinical case, a patient with pSjD-MN received dual BAFF/APRIL inhibition. The patient showed resolution of classical symptoms, normalization of immunological parameters, and complete recovery from proteinuria, hypoalbuminemia, and renal dysfunction (162). These findings collectively highlight the pivotal role of the BAFF/APRIL pathway in driving both systemic and renal immune pathology in pSjD. However, the clinical translation of molecular therapies remains limited due to insufficient evidence. Well-designed trials with kidney-specific primary endpoints are urgently needed to establish their renal protective effects.

Future research on the pathogenesis of renal impairment in pSjD should prioritize the following areas: 1) identifying key immune cell subsets and specific autoantibodies that drive renal injury; 2) discovering blood- or urine-based biomarkers for early detection of kidney involvement; 3) developing targeted therapies to control localized renal inflammation while minimizing systemic side effects; and 4) rigorously evaluating the efficacy and safety of novel interventions in clinical studies. The ultimate aim is to enable early diagnosis and precise treatment of pSjD-related renal damage, thereby improving patient outcomes.
